# Association between single, dual, poly use of tobacco products and smoking cessation in Korean adult smokers

**DOI:** 10.18332/tpc/214782

**Published:** 2026-01-27

**Authors:** Heajung Lee, Jaeyong Shin, Jae Woo Choi

**Affiliations:** 1 Department of Statistics and Data Science, Yonsei University, Seoul, Republic of Korea; 2 Department of Preventive Medicine, College of Medicine, Yonsei University, Seoul, Republic of Korea; 3 National Health Insurance Service, Wonju-si, Republic of Korea

**Keywords:** smoking cessation, combustible cigarette, electronic cigarette, heated tobacco product

## Abstract

**INTRODUCTION:**

This study examined the association between smoking cessation and different usage combinations of three tobacco products (combustible cigarette [CC], electronic cigarette [EC], heated tobacco product [HTP]) in Korean adults.

**METHODS:**

We analyzed repeated data from the Korea Health Panel Survey (KHPS), which consisted of nationally representative samples. A total of 1380 Korean adults participated in the study. The outcome of interest was whether the participant succeeded in quitting smoking all types of tobacco products. Participants were classified according to whether they smoked any of the three tobacco products (CCs and/or ECs and/or HTPs) based on their self-reported responses.

**RESULTS:**

A total of 211 participants had quit smoking during the follow-up period. After adjusting for potential confounding factors, the adjusted odds ratio (AOR) for smoking cessation was 3.15 (95% CI: 1.66–5.95), and 1.81 (95% CI: 0.63–5.21) for participants who currently smoke only HTPs (HTP-only user) and participants who currently vape only ECs (EC-only user), respectively, compared with participants who currently smoke only CCs (CC-only user). There was no significant association between dual or triple smoking and smoking cessation.

**CONCLUSIONS:**

HTP-only users had a statistically significant association with smoking cessation, with higher odds of quitting smoking within two years compared to CC-only users. Further studies with a large sample are required to validate our results considering a small number of participants in the comparison groups in this study.

## INTRODUCTION

Smoking tobacco products is one of the main factors contributing to early death or the onset of serious diseases, such as cardiovascular diseases, chronic respiratory diseases, and cancer^1^. The annual number of deaths due to smoking is estimated to be approximately eight million worldwide^1^. Tobacco use imposes a substantial burden on smoking-related healthcare costs on the global economy^2^. Hence, smoking cessation has been reported to have significant benefits in reducing the risk of tobacco-attributable mortality or morbidity^3^.

Electronic cigarettes (ECs) are battery-powered smoking devices that produce nicotine aerosols, and EC users inhale nicotine-containing vapors^4^. Previous studies have reported that vaping ECs showed significantly lower concentrations of tobacco-specific toxic biomarkers, such as tobacco-specific nitrosamines, polycyclic aromatic hydrocarbons, volatile organic compounds, or nicotine, than smoking combustible cigarettes (CCs)^5,6^. But, the effectiveness of ECs in improving health outcomes compared with CCs remains controversial^7^. In particular, there is conflicting evidence regarding the association between ECs and smoking cessation.

Several issues faced in previous studies on the association between ECs and smoking cessation require further exploration. First, most experimental or observational studies were conducted with participants from the United States (US) or Europe^8,9^. Considering that Asians are responsible for an increase in global tobacco sales and have different purchasing characteristics, studies of Asian populations need to be conducted^10^. Additionally, studies reflecting the diverse patterns of tobacco usage attributed to the advent of novel tobacco products are lacking. As an example, the global market value of heated tobacco products (HTPs), a type of novel tobacco product, is expected to reach approximately US$70 billion by 2027, which is 7-fold higher than its value in 2020^11^. Only a few studies have examined the difference in smoking cessation between combustible cigarette users and HTP users^12^. Previous studies involving HTP users were conducted in East Asian countries where HTPs are rapidly increasing their market share^12^; however, these studies investigated how different tobacco products are associated with the intention to quit smoking or quitting attempts, not successful quitting. As there is previously reported evidence of an insignificant association between intention to quit or quitting attempts and successful quitting^13^, research on smoking cessation among smokers using different types of tobacco products needs to be conducted. Also, previous findings have shown that psychological factors such as stress or depression are highly associated with smoking cessation^14^, but studies considering these mental factors are scant.

Therefore, this study aimed to examine the association between the combination of three types of tobacco products, CCs and/or ECs and/or HTPs, and smoking cessation among Korean adults, to understand the similarities or dissimilarities in single/dual/poly users in terms of their association with smoking cessation. Also, this study aimed to compare vaping ECs with smoking HTPs in their association with smoking cessation.

## METHODS

### Data source and study population

This study used the Korea Health Panel Survey (KHPS) database provided by the Korea institute for Health and Social Affair and the National Health Insurance Service. The KHPS database contains information on health behaviors or health status of Korean representative household members. Individuals for the KHPS were randomly selected using a two-stage stratified cluster sampling method, and samples are representative of the entire Korean population. The KHPS database covers demographics, socioeconomic status, substance use such as tobacco use, physical or mental health status, and healthcare cost. This study used KHPS data from 2019 to 2021 (KHPS version 2.2).

**Figure 1:**
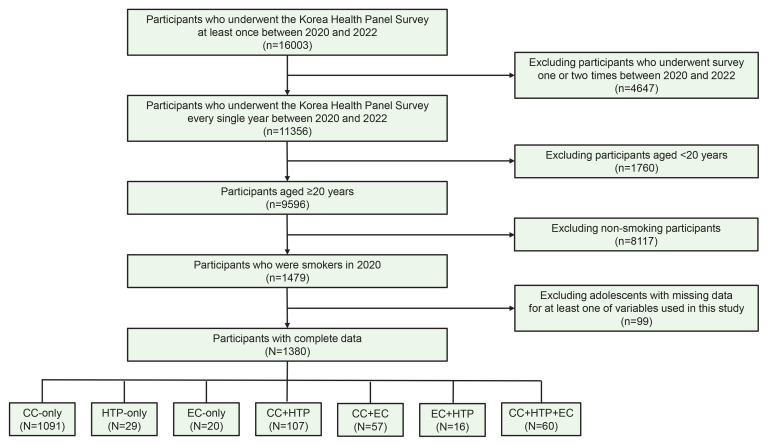
Flowchart of selection of study participants

Since KHPS database started to collect information on ECs or HTPs from 2019, we selected participants who participated in the KHPS at least once between 2019 and 2021. From 16003 eligible participants, we excluded 4647 participants who did not participate in the KHPS for the third consecutive year (2019–2021) in order to enable longitudinal data analysis. From 11356 eligible participants, we excluded 1760 participants aged <20 years in order to restrict our interest only to adults. From 9596 eligible participants, we excluded 8117 non-smokers or former smokers at baseline year (2019) in order to restrict our interest only to current smokers at baseline. From 1479 eligible participants, we excluded 99 participants who have missingness in their responses for at least one of questions mainly analyzed in this study. Finally, 1380 adults were included in this study. The flowchart of the inclusion/exclusion criteria of study participants is shown in Figure 1.

### Measures

*Tobacco product use*

The independent variable in this study was tobacco product use type. To categorize participants according to tobacco product use type, we examined responses to three respective questions (question on CC smoking, question on EC smoking, question on HTP smoking) at the baseline year, and classified participants into current smokers or non-current smokers (non-smokers, former smokers). In case of CCs, participants were asked whether they currently smoke CCs with four possible response options (currently smoke CCs every day, currently smoke CCs occasionally, smoked CCs in the past but not currently smoke, never smoked CCs). We defined participants who answered that they currently smoke CCs occasionally or every day as current CC smokers, and those who answered that they have never smoked or have smoked only in the past as non-current CC smokers. In case of ECs, participants were asked whether they have smoked ECs in the recent one month with two possible response options (yes, no). We defined participants who answered that they have smoked ECs as current EC smokers, and those who answered that they have not smoked ECs as non-current EC smokers. In case of HTPs, participants were asked whether they have smoked HTPs in the recent one month with two possible response options (yes, no). Pictures of HTPs with brand names were presented in the questionnaire to minimize participants’ confusion between ECs and HTPs. We defined participants who answered that they have smoked HTPs as current HTP smokers, and those who answered that they have not smoked HTPs as non-current HTP smokers.

Considering various usage combinations of tobacco products (CCs and/or ECs and/or HTPs), participants were categorized into following groups: 1) participants who currently smoke CCs solely (CC-only user), 2) participants who currently smoke ECs solely (EC-only user), 3) participants who currently smoke HTPs solely (HTP-only user), 4) participants who concurrently smoke both CCs and ECs (CC+EC dual user), 5) participants who concurrently smoke both CCs and HTPs (CC+HTP dual user), 6) participants who concurrently smoke both ECs and HTPs (EC+HTP dual user), and 7) participants who concurrently smoke all of CCs, ECs, HTPs (CC+HTP+EC triple user). CC-only users, HTP-only users, EC-only users, CC+HTP dual users, CC+EC dual users, EC+HTP dual users, and CC+HTP+EC triple users made up 79.1% (n=1091), 2.1% (n=29), 1.5% (n=20), 7.8% (n=107), 4.1% (n=57), 1.2% (n=16), and 4.4% (n=60) of all study participants, respectively.

*Smoking cessation*

The dependent variable in this study was whether participants succeeded in quitting smoking all types of tobacco products within two years. We assessed whether a participant was a current smoker or non-concurrent smoker at two different time points (after one year from baseline, after two years from baseline) to check if participants quit smoking within two years or not. We defined participants who were any of CC current smokers, EC current smokers, HTP current smokers as current smokers. Participants who were none of CC current smokers, EC current smokers, HTP current smokers were defined as non-current smokers. Of all participants who were current smokers at the baseline, participants who turned into non-current smokers within two years were classified as participants who succeeded in smoking cessation.

*Covariates*

We considered age, sex, marital status, education level, region, household income, body mass index (BMI), regular exercise, drinking level, chronic disease, perceived stress level, depression, anxiety answered by participants at baseline as potential confounding factors. We computed age at baseline, and then categorized into five groups (20–29, 30–39, 40–49, 50–59, and ≥60 years). Marital status was categorized into three groups (single, married, separated/widowed/divorced). Education level was categorized into three groups (middle school or lower, high school, college or higher). Administrative districts in Korea are currently divided into ‘Metropolitan city’, ‘Dong (neighborhood)’, ‘Myeon (district)’, or ‘Eup (town)’ based on the population size. Considering this fact, region was classified into following three groups: big city (‘Metropolitan city’), small and medium-sized city (‘Dong’), rural (‘Eup’ or ‘Myeon’). Household income was categorized into four groups according to the quartile values of household total income (Q1–Q4). BMI (kg/m^2^) was classified into following five groups: <18.5 (underweight), 18.5–23.0 (normal weight), 23.0–25.0 (overweight), 25.0–30.0 (class I obese), and ≥30 (class II obese), based on the World Health Organization’s recommendations for Asian populations^15^. Regular exercise was dichotomized according to whether participants regularly exercised (yes) or not (no) during the past year. Drinking level was stratified into four groups based on drinking frequency during the past year as follows: never drinker (none), 1–4 times per month (light), 2–6 times per week (moderate), and almost every day (heavy). The presence of chronic disease was assessed by asking participants about their history of chronic disease including hypertension, diabetes mellitus, cardiocerebrovascular disease, liver disease, chronic lower respiratory disease, musculoskeletal disorders, thyroid dysfunctions, malignant neoplasm, mental disorders, cognitive disorders, or chronic renal failure. In terms of stress level, respondents were asked to answer their perceived daily level of stress in one of four levels (low, moderate, high, very high). Depression was dichotomized according to whether participants felt considerable sadness or unhappiness that had a disruptive influence on their daily lives (yes) or not (no) for more than two consecutive weeks during past one year. Anxiety was dichotomized according to whether participants continuously felt considerable anxiety that had a disruptive influence on their daily lives (yes) or not (no) for more than six months during past one year.

### Statistical analysis

Regarding baseline characteristics of study participants according to tobacco product use type, variables were presented as the number of participants with corresponding percentages. chi-squared test was used to test for statistically significant difference among groups. To longitudinally examine how tobacco product use type is associated with smoking cessation, we used generalized estimating equation (GEE) models. GEE models have been developed for analyzing repeated measurements using generalized linear models in which the correlation structure is specified for clusters of observations^16^. GEE models enable to estimate population-averaged risk difference among groups, and also enable an unbiased estimate for coefficients corresponding to fixed-effect variables, even if the correlation structure is misspecified. GEE models in this study assumed binomial distribution with a logit link function, and an exchangeable working correlation structure. We adjusted for age, sex, marital status, education level, region, household income, BMI, regular exercise, drinking level, chronic disease, perceived stress level, depression, and anxiety at baseline. Results for association between tobacco product use type and smoking cessation were represented as estimates for odds ratio (OR) and corresponding 95% confidence interval (CI). A two-sided p<0.05 was considered statistically significant. All statistical analyses were performed using SAS software (version 9.4, SAS Institute, Cary, NC, USA).

## RESULTS

### Baseline characteristics

Among 1380 participants, CC-only users, HTP-only users, EC-only users, CC+HTP dual users, CC+EC dual users, EC+HTP dual users, and CC+HTP+EC triple users made up 79.1% (n=1091), 2.1% (n=29), 1.5% (n=20), 7.8% (n=107), 4.1% (n=57), 1.2% (n=16), and 4.4% (n=60) of all study participants, respectively. The number of participants that quit smoking within two years was 211, corresponding to 15.3% of all participants. Table 1 represents the baseline characteristics of study participants according to tobacco product use type. Adults aged ≥60 years comprised the highest proportion among all participants, and the percentage of the males was 89.9%. In terms of marital status or education level, 67.5% of the total participants were married, and 38.1% of the total participants were high school graduates. When it comes to sociodemographic or socio-economic factors, 42.4% lived in metropolitan cities, and 26.9% belonged to the lowest household income group. Out of all participants, 37.9% were in normal weight group. Regarding lifestyle factors, 44.0% regularly exercised, and 32.3% were moderate alcohol drinkers. In terms of disease or illness, 42.7% had chronic disease, 45.9% experienced moderate stress level, 8.4% felt substantial depressive feelings, and 5.7% had severe anxiety.

**Table 1:** Baseline characteristics of study participants according to tobacco product use type

*Characteristics*	*Total*	*Tobacco product use type*							p*
		*CC-only*	*HTP-only*	*EC-only*	*CC+HTP*	*CC+EC*	*EC+HTP*	*CC+HTP+EC*	
**Total**, n	1380	1091	29	20	107	57	16	60	
**Age** (years)									<0.001
20–29	87 (6.3)	58 (5.3)	2 (6.9)	3 (15.0)	9 (8.4)	6 (10.5)	-	9 (15.0)	
30–39	219 (15.9)	128 (11.7)	6 (20.7)	6 (30.0)	30 (28.0)	22 (38.6)	7 (43.8)	20 (33.3)	
40–49	300 (21.7)	203 (18.6)	11 (37.9)	5 (25.0)	40 (37.4)	15 (26.3)	6 (37.5)	20 (33.3)	
50–59	301 (21.8)	260 (23.8)	5 (17.2)	3 (15.0)	18 (16.8)	5 (8.8)	3 (18.8)	7 (11.7)	
≥60	473 (34.3)	442 (40.5)	5 (17.2)	3 (15.0)	10 (9.4)	9 (15.8)	-	4 (6.7)	
**Sex**									0.082
Male	1240 (89.9)	966 (88.5)	28 (96.6)	19 (95.0)	100 (93.5)	53 (93.0)	15 (93.8)	59 (98.3)	
Female	140 (10.1)	125 (11.5)	1 (3.5)	1 (5.0)	7 (6.5)	4 (7.0)	1 (6.3)	1 (1.7)	
**Marital status**									0.002
Single	243 (17.6)	175 (16.0)	4 (13.8)	4 (20.0)	23 (21.5)	18 (31.6)	2 (12.5)	17 (28.3)	
Married	931 (67.5)	731 (67.0)	23 (79.3)	15 (75.0)	76 (71.0)	33 (57.9)	14 (87.5)	39 (65.0)	
Separated/widowed/divorced	206 (14.9)	185 (17.0)	2 (6.9)	1 (5.0)	8 (7.5)	6 (10.5)	-	4 (6.7)	
**Education level**									<0.001
Middle school or lower	331 (24.0)	312 (28.6)	4 (13.8)	2 (10.0)	5 (4.7)	4 (7.0)	-	4 (6.7)	
High school	526 (38.1)	435 (39.9)	8 (27.6)	9 (45.0)	34 (31.8)	20 (35.1)	1 (6.3)	19 (31.7)	
College or higher	523 (37.9)	344 (31.5)	17 (58.6)	9 (45.0)	68 (63.6)	33 (57.9)	15 (93.8)	37 (61.7)	
**Region**									<0.001
Rural	301 (21.8)	270 (24.8)	2 (6.9)	2 (10.0)	13 (12.2)	8 (14.0)	2 (12.5)	4 (6.7)	
Small and medium-sized city	494 (35.8)	373 (34.2)	14 (48.3)	5 (25.0)	49 (45.8)	24 (42.1)	6 (37.5)	23 (38.3)	
Metropolitan city	585 (42.4)	448 (41.1)	13 (44.8)	13 (65.0)	45 (42.1)	25 (43.9)	8 (50.0)	33 (55.0)	
**Household income**									<0.001
Q1	371 (26.9)	344 (31.5)	5 (17.2)	2 (10.0)	9 (8.4)	9 (15.8)	-	2 (3.3)	
Q2	360 (26.1)	286 (26.2)	9 (31.0)	5 (25.0)	25 (23.4)	16 (28.1)	6 (37.5)	13 (21.7)	
Q3	328 (23.8)	240 (22.0)	6 (20.7)	7 (35.0)	33 (30.8)	19 (33.3)	2 (12.5)	21 (35.0)	
Q4	321 (23.3)	221 (20.3)	9 (31.0)	6 (30.0)	40 (37.4)	13 (22.8)	8 (50.0)	24 (40.0)	
**BMI** (kg/m^2^)									<0.001
Underweight	40 (2.9)	39 (3.6)	-	-	1 (0.9)	-	-	-	
Normal	523 (37.9)	439 (40.2)	15 (51.7)	3 (15.0)	35 (32.7)	15 (26.3)	2 (12.5)	14 (23.3)	
Overweight	357 (25.9)	284 (26.0)	6 (20.7)	7 (35.0)	26 (24.3)	10 (17.5)	4 (25.0)	20 (33.3)	
Class I obesity	401 (29.1)	287 (26.3)	8 (27.6)	10 (50.0)	39 (36.5)	28 (49.1)	9 (56.3)	20 (33.3)	
Class II obesity	59 (4.3)	42 (3.9)	-	-	6 (5.6)	4 (7.0)	1 (6.3)	6 (10.0)	
**Regular exercise**									0.767
No	773 (56.0)	610 (55.9)	20 (69.0)	9 (45.0)	59 (55.1)	33 (57.9)	8 (50.0)	34 (56.7)	
Yes	607 (44.0)	481 (44.1)	9 (31.0)	11 (55.0)	48 (44.9)	24 (42.1)	8 (50.0)	26 (43.3)	
**Drinking level**									0.002
None	326 (23.6)	270 (24.8)	10 (34.5)	-	20 (18.7)	11 (19.3)	2 (12.5)	13 (21.7)	
Light	455 (33.0)	351 (32.2)	8 (27.6)	11 (55.0)	46 (43.0)	17 (29.8)	5 (31.3)	17 (28.3)	
Moderate	445 (32.3)	333 (30.5)	6 (20.7)	9 (45.0)	36 (33.6)	26 (45.6)	8 (50.0)	27 (45.0)	
Heavy	154 (11.2)	137 (12.6)	5 (17.2)	-	5 (4.7)	3 (5.3)	1 (6.3)	3 (5.0)	
**Chronic disease**									<0.001
No	791 (57.3)	578 (53.0)	20 (69.0)	15 (75.0)	81 (75.7)	40 (70.2)	12 (75.0)	45 (75.0)	
Yes	589 (42.7)	513 (47.0)	9 (31.0)	5 (25.0)	26 (24.3)	17 (29.8)	4 (25.0)	15 (25.0)	
**Perceived stress level**									0.138
Low	232 (16.8)	196 (18.0)	5 (17.2)	1 (5.0)	15 (14.0)	4 (7.0)	-	11 (18.3)	
Moderate	633 (45.9)	507 (46.5)	13 (44.8)	10 (50.0)	46 (43.0)	29 (50.9)	8 (50.0)	20 (33.3)	
High	421 (30.5)	320 (29.3)	8 (27.6)	6 (30.0)	41 (38.3)	18 (31.6)	7 (43.8)	21 (35.0)	
Very high	94 (6.8)	68 (6.2)	3 (10.3)	3 (15.0)	5 (4.7)	6 (10.5)	1 (6.3)	8 (13.3)	
**Depression**									0.434
No	1264 (91.6)	997 (91.4)	29 (100)	18 (90.0)	100 (93.5)	51 (89.5)	16 (100.0)	53 (88.3)	
Yes	116 (8.4)	94 (8.6)	-	2 (10.0)	7 (6.5)	6 (10.5)	-	7 (11.7)	
**Anxiety**									0.718
No	1301 (94.3)	1028 (94.2)	29 (100)	19 (95.0)	101 (94.4)	53 (93.0)	16 (100.0)	55 (91.7)	
Yes	79 (5.7)	63 (5.8)	-	1 (5.0)	6 (5.6)	4 (7.0)	-	5 (8.3)	

Data are expressed as number of participants (%). BMI: body mass index. CC: combustible cigarette. EC: electronic cigarette. HTP: heated tobacco product.

* Chi-squared test.

Compared to CC-only users, HTP-only/EC-only users or dual/triple users were more likely to be aged <50 years, and more likely to be college educated. Also, HTP-only/EC-only users or dual/triple users were more likely to live in cities and belonged to the higher income group. EC-only users or dual/triple users were less likely to be in the normal weight group. In terms of health-related factors, EC-only users or dual/triple users were more likely to be light or moderate alcohol drinkers, and HTP-only/EC-only users or dual/triple users were less likely to have chronic disease.

### Association between tobacco product use type and smoking cessation

Table 2 presents OR and 95% CI for smoking cessation associated with tobacco product use type in adults. After adjusting for possible confounding factors, AOR for smoking cessation was 3.15 (95% CI: 1.66–5.95; p<0.001), 1.81 (95% CI: 0.63–5.21; p=0.273), 0.89 (95% CI: 0.54–1.46; p=0.642), 0.86 (95% CI: 0.37–1.97; p=0.718), 1.10 (95% CI: 0.29–4.07; p=0.892), and 0.60 (95% CI: 0.27–1.32; p=0.201) for HTP-only users, EC-only users, CC+HTP dual users, CC+EC dual users, EC+HTP dual users, and CC+HTP+EC triple users, respectively, compared to CC-only users. Although HTP-only users were relatively small in number, OR for smoking cessation was significantly higher only in HTP-only users, compared to CC-only users. There was no significant association between EC-only smoking and smoking cessation. Similarly, there was no significant association between dual or triple smoking and smoking cessation.

**Table 2:** The odds ratio of smoking cessation in relation to tobacco product use type

*Variables*	*Unadjusted Model*		*Adjusted Model 1*		*Adjusted Model 2*	
	*OR (95% CI)*	*p*	*AOR (95% CI)*	*p*	*AOR (95% CI)*	*p*
CC-only (ref.)	1.00		1.00		1.00	
HTP-only	2.37 (1.30–4.31)	0.005	3.02 (1.64–5.54)	<0.001	3.15 (1.66–5.95)	<0.001
EC-only	1.58 (0.54–4.61)	0.401	1.88 (0.64–5.49)	0.248	1.81 (0.63–5.21)	0.273
CC+HTP dual	0.83 (0.53–1.31)	0.421	0.96 (0.58–1.59)	0.882	0.89 (0.54–1.46)	0.642
CC+EC dual	0.85 (0.40–1.79)	0.665	0.93 (0.42–2.06)	0.849	0.86 (0.37–1.97)	0.718
EC+HTP dual	1.04 (0.30–3.63)	0.954	1.28 (0.34–4.81)	0.713	1.10 (0.29–4.07)	0.892
CC+HTP+EC triple	0.59 (0.30–1.18)	0.134	0.66 (0.31–1.41)	0.286	0.60 (0.27–1.32)	0.201

GEE model was constructed to examine the odds of smoking cessation according to tobacco product use type. AOR: adjusted odds ratio. Model 1 was adjusted for age and sex. Model 2 was adjusted for age, sex, marital status, education level, region, household income, BMI, regular exercise, drinking level, chronic disease, perceived stress level, depression, and anxiety. BMI: body mass index. CC: combustible cigarette. EC: electronic cigarette. GEE: generalized estimating equation. HTP: heated tobacco product.

Participants were asked whether they have attempted to quit smoking during the past year, and 238 participants, corresponding to 17.3% of all participants, answered that they had past quit attempts. To determine if our analysis was biased by inclusion of participants with past quit attempts, we conducted a sensitivity analysis by excluding participants with past quit attempts. The sensitivity analysis showed that OR for smoking cessation was significantly higher in HTP-only users (Supplementary file Table 1), compared to CC-only users. This sensitivity analysis demonstrated consistency and robustness of our results to selection bias regarding heterogeneity between participants with and without past quit attempts.

## DISCUSSION

This study examined the association between smoking cessation and different types of tobacco products. When we classified smokers according to whether they smoked three types of tobacco products (CCs and/or ECs and/or HTPs), our results showed that HTP-only users were the only ones who had a significant association with smoking cessation compared to CC-only users. HTP-only users had a statistically significant association with smoking cessation, with higher odds of quitting smoking within two years compared to CC-only users.

Our results for HTP-only users are consistent with previous studies^17,18^; however, two cross-sectional studies among Korean adults have reported inconsistent results^19,20^. One study suggested that the odds of being a former CC smoker was significantly lower among current HTP-only users, who had ever smoked CCs in the past, than current CC-only users^20^. Another study compared past quit attempts or future cessation plans between HTP-only users and CC-only users, and suggested that HTP-only users have significantly fewer past attempts for more than one day or fewer smoking cessation plans within a month than CC-only users^19^. This inconsistency between this study and others may be attributable to difference in outcome of interest when it comes to staging of smoking cessation (final stage vs contemplation or preparation stage) or targeted tobacco product type for cessation (quitting all kinds of tobacco products vs quitting CCs). In the case of EC-only users, previous meta-analysis results were consistent with our findings for insignificant association between EC-only use and smoking cessation^21-23^. But some previous studies suggested that EC use had significant association with smoking cessation^17,24-27^. These studies commonly included adult smokers in the US or Europe, and also assessed short-term smoking cessation, such as quitting within past 30 days or one year, based on self-reported abstinence. Individual studies differed in several study design factors, such as study participant (only smokers with motivation to quit vs smokers with/without motivation to quit), reference group (never EC users vs never/former EC users), sample size, or list of confounding factors such as smoking intensity or nicotine dependence.

In the case of dual or triple users, most previous studies showed results consistent with ours^19,28^; however, two cross-sectional studies among Korean adults showed dissimilar results^20,29^. One study reported that CC+EC dual users or triple users had significantly higher prevalence ratio of attempts to quit CCs within the past year than CC-only users^29^. This study was similar to our study in that it reported insignificant difference in prevalence ratio of attempts to quit CCs between CC+HTP dual users and CC-only users. Another study reported that the odds of being a former CC smoker was significantly lower for current EC+HTP dual users than for current CC-only users^20^. The inconsistency among studies on smoking cessation among dual or triple tobacco users may be due to opposing points of view regarding multiple tobacco users. Those who regard dual or triple users as smokers with high nicotine dependence claim that strong nicotine dependence may prevent multiple tobacco users from successfully quitting smoking^20^. Some dual or triple users can represent smokers who are in the middle of switching from one tobacco product to another^30^. In terms of dual users’ frequency or intensity of smoking, one previous study reported that dual users did not reduce their number of cigarettes during the study period, and concluded that there is no significant difference in the likelihood of quitting smoking between dual smokers and CC-only smokers after 12 months^31^. Similarly, one previous study investigating profiles of dual users revealed that there was significant difference in reasons for dual use between predominantly CC smokers, who have daily CC use and non-daily EC use, and predominantly EC smokers, who have daily EC use and non-daily CC use^32^. The study reported that predominantly EC smokers were more likely to use ECs to help quit smoking whereas predominantly CC smokers were more likely to use ECs to compensate for their decreased nicotine intake^32^. In light of this, degree of similarity in the proportion of predominantly CC smokers and predominantly EC smokers among studies can be one possible factor explaining consistent or inconsistent results in dual users’ smoking cessation compared to CC-only smokers. But, further studies on the characteristics of multiple tobacco users are required to determine the association between multiple tobacco use and smoking cessation more thoroughly.

This study’s strength lies in finding of significant association between HTP-only use and cessation within two years by examining how diverse usage combinations of tobacco products is associated with cessation in Asian adult smokers. The greater abstinence effect of the addition of alternatives to traditional CCs to standard smoking cessation counseling over counseling alone was shown in one recent randomized clinical trial^33^. But, the validity or reliability of the use of alternatives to CCs as an aid to smoking cessation still needs to be discussed considering conflicting results. The controversy surrounding the role of alternatives to CCs as a smoking cessation aid is linked to different regulatory environments toward alternatives to traditional CCs among countries. For example, ECs have been officially accepted as a smoking cessation aid in the United Kingdom, however, the US Food and Drug Administration has not approved it^34^. It is important to note that the introduction of well-organized EC regulations is significantly associated with improving cessation rates^35^, regardless of whether a country has endorsed ECs as a smoking cessation aid or not. Most cessation policies or programs in Korea still focus on reducing the number of CC smokers, which lacks timeliness considering diversification of tobacco products. In addition, the management or disclosure system of information on tobacco products needs to be improved in a way that consumers can obtain more accurate information about various tobacco products. Therefore, we suggest that our findings can be used as a basis for improving the timeliness and concreteness of current Korea tobacco control policies or smoking cessation programs.

### Limitations

There are several limitations in this study. First, the causality between tobacco product use and smoking cessation could not be determined owing to the retrospective nature of this study. Second, our definition for smoking-related variables was based on self-reported responses to questionnaires, which lacked information on biological markers of smoke exposure. Previous studies revealed high reliability of self-reported data on adults’ smoking behavior^17,22^ or high level of agreement between self-reported response and biological measurement in smokers^36^. Potential for bias related to self-reported data cannot be ruled out. Further studies based on smokers’ biological data are needed to validate the robustness of our findings. Third, duration of tobacco product use or motivation for switching between tobacco products could not be taken into account in our analysis due to the unavailability of corresponding data. Adjustment to more in-depth smoking information is required in future studies to demonstrate their association with smoking cessation. Fourth, this study included data collected during the Corona Virus Disease 2019 pandemic. Therefore, our results may have been affected by the extraordinary nature of the pandemic. Finally, our findings among Korean adults cannot be extrapolated to populations with different smoking patterns, such as individuals of different ethnicities or nations. Also, further studies with a large sample are required for generalization of our findings, considering a relatively small number of participants in the comparison groups (HTP-only users, EC-only users, CC+HTP dual users, CC+EC dual users, EC+HTP dual users, CC+HTP+EC triple users), compared to the reference group (CC-only users), in this study.

## CONCLUSIONS

We demonstrated that HTP-only use has a significant association with smoking cessation, with higher odds of quitting smoking within two years compared to CC-only use. Our results can be helpful in developing smoking cessation programs customized to smokers’ current tobacco usage pattern, such as suggesting sole HTP use as an aid for smoking cessation or warning against dual/triple use of different types of tobacco products. Any decision-making based on our results needs considerable attention due to the quite small number of participants in the comparison groups in this study. Further studies with a large sample are required to validate our results on the association between smoking cessation and different usage combinations of tobacco products.

## Supplementary Material



## Data Availability

The data supporting this research are available from the following source: https://www.khp.re.kr:444/eng/data/data.do.
