# Supplementary material for: Association between single, dual, poly use of tobacco products and smoking cessation in Korean adult smokers
Source: Tob Prev Cessat. 2026 Jan 27;12:10.18332/tpc/214782. doi: 10.18332/tpc/214782 (PMC12838446; doi:10.18332/tpc/214782)
Supplement: Supplementary file 1 [file TPC-12-04-s1.pdf]

Supplementary Table 1. Sensitivity analysis evaluating the odds ratio of smoking cessation in relation to tobacco product use type by excluding participants with past quit smoking attempts

	Unadjusted Model		Adjusted Model 1		Adjusted Model 2	
Tobacco product use type	OR (95% CI)	*p*-value	OR (95% CI)	*p*-value	OR (95% CI)	*p*-value
CC-only	1.00		1.00		1.00	
HTP-only	1.66 (0.91, 3.03)	0.099	1.95 (1.08, 3.52)	0.026	1.82 (1.03, 3.22)	0.040
EC-only	2.19 (0.63, 7.63)	0.217	2.43 (0.69, 8.60)	0.169	2.44 (0.69, 8.66)	0.168
CC+HTP dual	0.82 (0.52, 1.30)	0.398	0.93 (0.56, 1.55)	0.776	0.86 (0.52, 1.42)	0.559
CC+EC dual	0.78 (0.35, 1.76)	0.551	0.79 (0.33, 1.90)	0.601	0.74 (0.29, 1.89)	0.528
EC+HTP dual	1.77 (0.61, 5.20)	0.296	2.15 (0.69, 6.72)	0.190	1.85 (0.62, 5.50)	0.268
CC+HTP+EC triple	0.74 (0.43, 1.27)	0.279	0.78 (0.43, 1.41)	0.407	0.68 (0.36, 1.28)	0.233

GEE model was constructed to examine the odds of smoking cessation according to tobacco product use type. Model 1 was adjusted for age and sex. Model 2 was adjusted for age, sex, marital status, education level, region, household income, BMI, regular exercise, drinking level, chronic disease, perceived stress level, depression, and anxiety.

BMI, body mass index; CC, combustible cigarette; CI, confidence interval; EC, electronic cigarette; GEE, generalized estimating equation; HTP, heated tobacco product; OR, odds ratio.

© 2026 Lee H. et al.
